# 11,12 and 14,15 epoxyeicosatrienoic acid rescue deteriorated wound healing in ischemia

**DOI:** 10.1371/journal.pone.0209158

**Published:** 2019-01-16

**Authors:** Katharina Sommer, Heike Jakob, Farsin Badjlan, Dirk Henrich, Johannes Frank, Ingo Marzi, Anna Lena Sander

**Affiliations:** 1 Department of Trauma, Hand and Reconstructive Surgery, Hospital of the Johann Wolfgang Goethe-University, Frankfurt am Main, Germany; 2 Department of Trauma, Hand and Reconstructive Surgery, Marienhausklinik St. Josef Kohlhof, Neunkirchen, Germany; Medical University Innsbruck, AUSTRIA

## Abstract

**Introduction:**

Epoxyeicosatrienoic acids (EETs) are able to enhance angiogenesis and regulate inflammation that is especially important in wound healing under ischemic conditions. Thus, we evaluated the effect of local EET application on ischemic wounds in mice.

**Methods:**

Ischemia was induced by cautherization of two of the three supplying vessels to the mouse ear. Wounding was performed on the ear three days later. Wounds were treated either with 11,12 or 14,15 EET and compared to untreated control and normal wounds. Epithelialization was measured every second day. VEGF, TNF-α, TGF-β, matrix metalloproteinases (MMP), tissue inhibitors of metalloproteinases (TIMP), Ki67, and SDF-1α were evaluated immunohistochemically in wounds on day 3, 6, and 9.

**Results:**

Ischemia delayed wound closure (12.8 days ± 1.9 standard deviation (SD) for ischemia and 8.0 days ± 0.94 SD for control). 11,12 and14,15 EET application ameliorated deteriorated wound healing on ischemic ears (7.6 ± 1.3 SD for 11,12 EET and 9.2 ± 1.4 SD for 14,15 EET). Ischemia did not change VEGF, TNF-α, TGF-β, SDF-1α, TIMP, MMP7 or MMP9 level significantly compared to control. Local application of 11,12 as well as 14,15 EET induced a significant elevation of VEGF, TGF-β, and SDF-1α expression as well as proliferation during the whole phase of wound healing compared to control and ischemia alone.

**Conclusion:**

In summary, EET improve impaired wound healing caused by ischemia as they enhance neovascularization and alter inflammatory response in wounds. Thus elevating lipid mediator level as 11,12 and 14,15 EET in wounds might be a successful strategy for amelioration of deranged wound healing under ischemia.

## Introduction

Wound healing is a highly regulated process that restores integrity of the skin. Failure of the orderly sequence of steps for wound closure leads to chronic wounds. Especially resolution of inflammation and angiogenesis are highly important in this process. Several systemic diseases as diabetes mellitus and arteriosclerosis promote the development of chronic wounds as they diminish blood supply that leads to a decreased oxygen pressure on the wound side. Thus, it has already been proposed, that enhancing neovascularisation as means to improve blood supply seems to be the key step to rescue deteriorated wound healing in chronic wounds [1]. Secondly, chronic wounds fail to resolve inflammation that is important to switch to proliferation and tissue formation stage. For this reason, regulation of inflammation in the wound side also seems to be a promising approach to enhance deteriorated wound healing in chronic wounds [2].

Epoxyeicosatrienoic acids (EETs) are lipid mediators derived of arachidonic acid by cytochrome P450 epoxygenases. They are known to regulate inflammation, angiogenesis, and vascular tone [3]. In our previous work we could already show that local application of 11,12 EET and 14,15 EET can accelerate wound healing by enhancing local neovascularisation under normal conditions in mice [4]. These effects are at least partially mediated by enhanced VEGF expression in regenerating tissues [4,3]. VEGF is known to be a key factor for mediating angiogenesis during the proliferative phase of wound healing [5]. Besides its potent pro-angiogenic properties VEGF also promotes endothelial cell activation and migration [6]. It has already been shown, that under ischemic conditions wounds display a higher expression of VEGF than normal [7]. Furthermore, exogenous administration of VEGF under hypoxic conditions additionally enhances angiogenesis and improves the survival of ischemic skin flaps [7]. Besides these facts, EETs also stimulate formation of collateral blood supply in ischemic tissues [8]

Furthermore, EETs have anti-inflammatory properties and enhance vasodilatation, cellular proliferation and migration. Panigrahy et al. could also demonstrate that EETs accelerate tissue growth and regeneration in different organs [3]. In an animal model of lung injury EETs diminish organ damage by protecting against the pro-apoptotic effects of TNF-α [9]. TNF-α exerts a double sided role during wound healing [14]. High concentration of this cytokine inhibit granulation tissue formation and suppresses the function of TGF-β that is highly important for proliferation. TGF-β also stimulated collagen production thus enhancing formation of extracellular matrix [10]. This is partly mediated by up-regulation of tissue inhibitors of metalloproteinases (TIMPs) that negatively regulate the function of matrix metalloproteinases (MMPs). Thus low concentrations of TNF-α promote collagen deposition [11–14,10]

MMPs are important for wound closure and reepithelialisation as they facilitate keratinocyte migration [15]. They also take part in activation and degradation of cytokines and are important for angiogenesis and scar formation [16]. During wound healing inhibition of soluble epoxide hydrolase that degrades EETs alters expression of matrix metalloproteinases and their inhibitor TIMP1 [17]. This could also be shown in *in vitro* experiments as EET treatment of cancer cells resulted in an up-regulation of MMP9 expression [18]. Treatment with 11,12 EET also enhances MMP activity in endothelial cells [19].

Besides these effects EETs also enhance SDF-1α expression in wounds [4]. SDF-1α is an important homing factor for stem and progenitor cells that take part in coordinating wound repair by cytokine production [20]. SDF-1α also promotes keratinocyte proliferation and migration *in vitro* which could partly explain its positive effect on wound repair [21].

In the following study, we examined the effect of local EET application on wounds under ischemic conditions and analyzed the effects on inflammation and neovascularization on the wound side.

## Material and methods

To analyze wound repair under ischemic conditions we used a model of ischemia on mouse ears that we combined with a creation of a wound on the dorsal side of the ear. Epithelialization of the wounds was measured *in vivo* in animals. For analysis of neovascularization, inflammation and proliferation expression of CD31, VEGF, TNF-α, TGF-β, MMP7, MMP9, SDF-1α, and Ki67 were measured immunohistochemically after sacrifice of the animals on day 3, 6, and 9 after wound creation.

### Animals

All animal experiments were performed in accordance with ethic guidelines of German law and approved by the Regierungspräsidium Darmstadt (Ethic Approval no. V54–19c20/15–F3/17).

Male hairless SKH-1 mice (weight 25–35 g; age 8–10 weeks) were obtained from Charles River Laboratories (Sulzfeld, Germany). Animals were housed in separate cages at 24 ˚C with light intervals of 12 h/day in airflow regulated rooms, and fed a balanced rodent diet with water *ad libitum*. For all surgical interventions the animals were anesthetized with intraperitoneal (i.p.) injection of 100μl solution containing 2.215mg of ketamine and 0.175mg of xylazine hydrochloride. For all wound measurements animals were anesthetized the same as for surgery. At the end of the experiments animals were euthanized by cervical dislocation [22].

### Induction of ischemia

Ischemia of the mouse ears was created by cauterization of all but one of the three to four neurovascular bundles that supply the ear leaving only the most anterior bundle for blood circulation as described previously [23]. This procedure was done three days prior to wound creation on the ears.

### Wound creation

Wounds were created on the dorsum of both ears three days after the induction of ischemia. To attain a standard size of the wounds a circular punch (diameter 2.25 mm) was used to incise a full thickness layer of ear skin down to the underlying cartilage, as described previously. After the punch incision, the skin was carefully removed from the underlying cartilage. Subsequently, the wound was covered with a small methylcellulose pad according to the treatment group with or without 11,12 EET or 14,15 EET (Cayman Chemical Company, Ann Arbor, Michigan, USA). Pads were manufactured by taking 200μl of 2,5% carboxymethylcellulose in PBS and adding either 200μl ethanol for control or 160μl ethanol and 40μl of 95% 11,12 EET or 98% 14,15 EET to the mixture. To obtain a pad the mixture was dried for 20 minutes on a hot plate at 37° Celsius on parafilm.

Finally, the entire ear was covered with a bioadhesive dressing (Opsite; Smith and Nephew Medical Ltd., Tuttlingen, Germany) [24,22].

### Wound epithelialization and wound closure measurements

The wound model on the mouse ear allows direct measurement of epithelialization of the wound as wound contraction that normally occurs in mice is impeded as the skin of the ear is directly attached to the underlying cartilage. Thus, epithelialization could directly be measured in living animals.

On the day of wounding (day 0) and subsequently every second day thereafter, wound epithelialization was measured by direct visualization and quantification using *in vivo* microscopy and computerized planimetry. Measurements were performed by placing the outstretched ear of the anesthetized animals on an acrylic glass platform on the stage of an intra-vital microscope (Carl Zeiss, Oberkochen, Germany). The microscope image was captured with a low light camera (DXC-390P, 3CCD color video camera; Sony, Tokyo, Japan) and transferred through a digital converter (ADVC-100; Canopus, Ruppach-Goldhausen, Germany). Digitalized images were analyzed using the ImageJ software (http://rsb.info.nih.gov/ij/download.html) by tracing the wound margin and calculating the area. The rate of wound-closure was expressed as the ratio of the wounded area at each time point divided by the original wound area at time 0.

Wound closure was determined as the day of complete epithelialization of the wound. Analysis was performed by an independent investigator [25].

### Evaluation of angiogenesis, inflammatory reaction, and proliferation in wounds

Wounded ears from killed animals were embedded in TissueTek (Sakura Finetek Europe, Zoeterwoude, The Netherlands) on day 2, 6, 10, and 16 after surgery and stored at –80°C. For immunohistochemical staining, wounds were cut to sections of 5μm thickness.

For analysis, the sections were submerged in acetone (–20°C, 10min) followed by 10 min 0.1% hydrogen peroxidase treatment. Sections were stained with primary antibodies directed against VEGF (Abcam, Cambridge, UK) for evaluation of angiogenesis, TNF-α, TGF-β, and SDF-1α (Abcam, Cambridge, UK) and MMP7, MMP9 and TIMP1 (Abbiotec, San Diego, USA) for wound cytokine expression, and Ki67 (Dako, Hamburg, Germany) for proliferation for 1h at RT. Primary antibodies were detected by HRP-AEC (Abcam, Cambridge, UK) staining according to the guidelines of the manufacturer. Sections were counterstained with hematoxylin and viewed at 100x magnification (Axio Observer; Carl Zeiss, Oberkochen, Germany). The microscope image was captured with a low light camera (AxioCam; Carl Zeiss, Oberkochen, Germany) and digitalized. Photographic images were analyzed using the ImageJ software (https://imagej.nih.gov/ij/). The staining of each section was standardized to the area scanned. The analysis was performed by an independent investigator [25].

### Statistical analysis

Data are presented as the mean ± SD. Statistical evaluation was performed with non-parametric Kruskal-Wallis test followed by a Dunn post-hoc test using a Bonferroni-Holm adjustment with Bias 10.0 (Epsilon-Verlag, Darmstadt, Germany) comparing the evaluated two groups at the same point of time. Values of p<0.05 were considered statistically significant. The number of samples examined is indicated by n.

## Results

### Wound closure and reepithelialisation process during wound healing

For evaluation of wound closure after induction of ischemia by ligation of 2 of the 3 supplying vessels to the mouse ear and effect of local 11,12 EET and 14,15 EET on ischemic ears, we monitored day of wound closure and the process of reepithelialisation throughout the wound healing process. For the later purpose, wound area was measured every second day after wounding of the ear.

**Fig 1:**
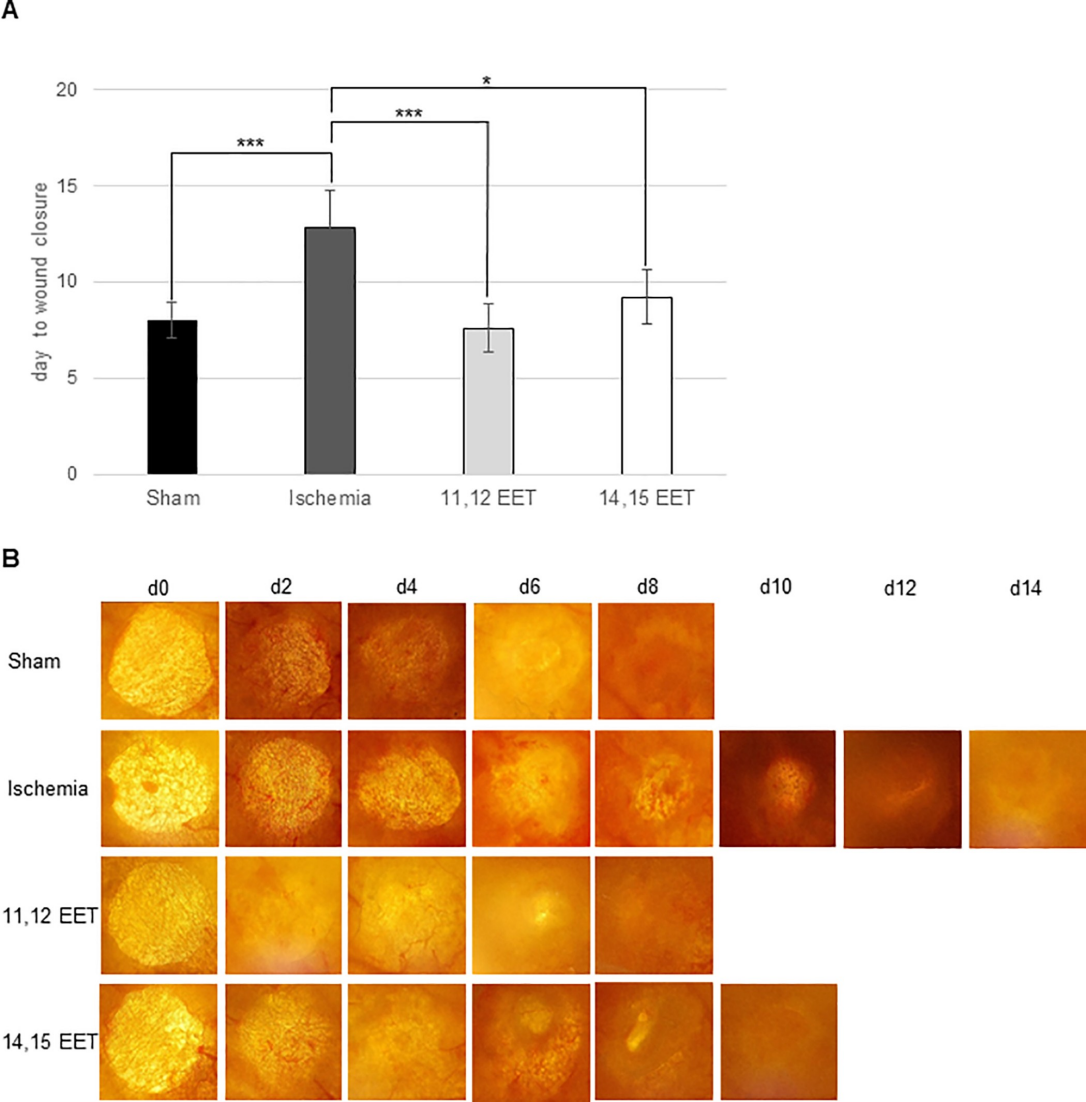
A Day of wound closure. Depicted is day of wound closure of controlwounds, ischemic wounds and ischemic wounds after treatment with 11,12 and 14,15 EET. B Representative in vivo pictures of wound healing taken every second day (data is shown as mean ± SD; *n = 10*). *p<0.05, **p<0.01, ***p<0.001.

Full reepithelialisation of the wound occurred in ischemic ears on day 12.8 ± 1.9 standard deviation (SD) whereas wounds of control mice closed on day 8.0 ± 0.94 SD. (Fig 1A).

Local application of EETs was able to rescue impaired wound healing after induction of ischemia. Wounds on 11,12 EET treated ischemic ears closed on day 7.6 ± 1.3 SD and 14,15 EET treated mice on day 9.2 ± 1.4 SD (Fig 1A). Thus, 11,12 EET showed a better potential for rescuing impaired wound healing after induction of ischemia than 14,15 EET though this finding was not significant (Fig 1A). Comparing non ischemic control and ischemic ears after 11,12 as well as 14,15 EET application no significant difference was observed.

**Fig 2:**
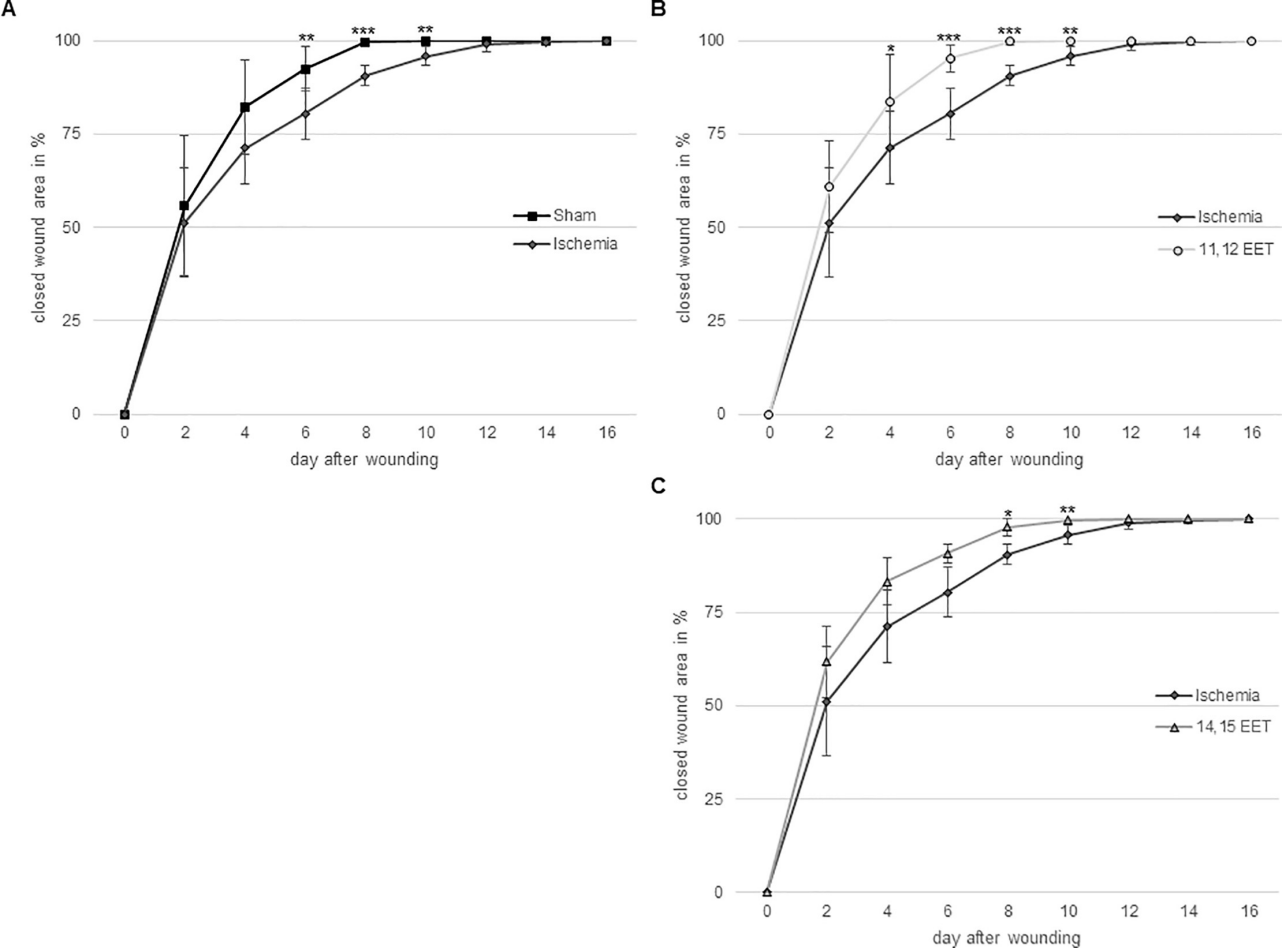
A Percentage of closed wound area from day 0–16 of ischemic and non-ischemic ears. B Percentage of closed wound area from day 0–16 of ischemic and 11,12 EET treated wounds. C Percentage of closed wound area from day 0–16 of ischemic and 14,15 EET treated wounds. (data is shown as mean ± SD; *n = 10*).*p<0.05, **p<0.01, ***p<0.001.

These results are well supported by measurement of closing wound area in vivo throughout the wound healing process. Reepithelialisation was delayed after day 6 up to day 10 after wounding in ischemic ears compared to control mice (Fig 2A). Application of 11,12 EET on ischemic ears significantly enhanced delayed reepithelialisation from day 4 up to day 10 (Fig 2B) and application of and 14,15 EET ameliorated wound healing from day 8 to day 10 compared to ischemic ears (Fig 2C). On day 6 and day 8 11,12 EET showed an even faster wound closure than application of 14,15 EET in accordance to the fact that 11,12 ETT also enhanced wound closure compared to 14,15 EET thought this finding was not significant. Furthermore, No significant difference in wound closure was seen when comparing control and 11,12 as well as 14,15 EET.

Summing up 11,12 EET as well as 14,15 EET were able to ameliorate delayed wound healing in ischemic wounds. Moreover 11,12 EET exhibited even a better potential for rescuing impaired wound healing than 14,15 EET.

### Evaluation of local neovascularisation in wounds

We evaluated neovascularisation in ischemic wounds and after treatment with EETs by measuring VEGF expression immunohistochemically.

**Fig 3:**
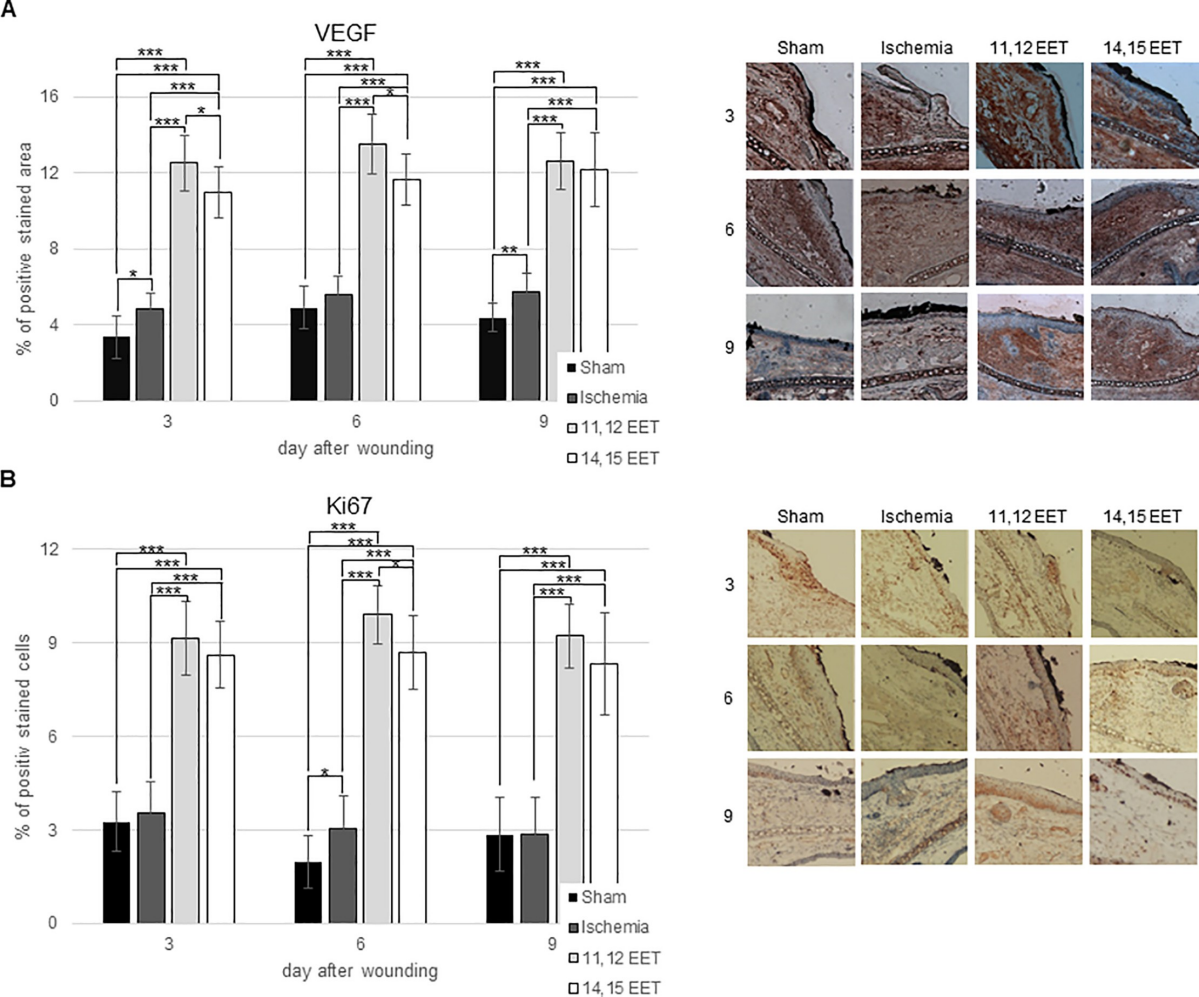
A Percentage of VEGF positive area on day 3, 6, and 9 after wounding ofcontrol, ischemic and 11,12 as well as 14,15 EET treated ischemic wounds. On the right representative pictures of immunohistological staining. B Percentage of Ki67 positive cells on day 3, 6 and 9 after wounding ofcontrol, ischemic and 11,12 as well as 14,15 EET treated ischemic wounds. On the right representative pictures of immunohistological staining (data is shown as mean ± SD; *n = 8*). *p<0.05, ***p<0.001.

Wound on ischemic ears seemed to express more VEGF on throughout the whole wound healing process compared to control though this finding was not significant (Fig 3A). Local treatment with 11,12 EET elevated VEGF expression in wounds on day 3, 6, and 9 compared to control and to non-treated ischemic wounds and 14,15 EET on day 3, 6, and 9 compared to control as well as on day 6 and 9 compared to ischemia alone (Fig 3A). Furthermore, 11,12 EET seemed to enhanced VEGF expression more than 14,15 EET treatment though this was not significant (Fig 3A). Thus, local EET treatment enhances neovascularization in ischemic wounds.

### Evaluation of local inflammation reaction in wounds

Local inflammatory reaction is a key step to wound healing. Thus local TNF-α and TGF-β expression was measured in ischemic and non-ischemic wounds as well as after treatment with 11,12 EET and 14,15 EET. Expression of MMP7 and MMP9 and their inhibitor TIMP1 were also evaluated immunohistochemically. Furthermore, SDF-1α was stained in wounds.

**Fig 4:**
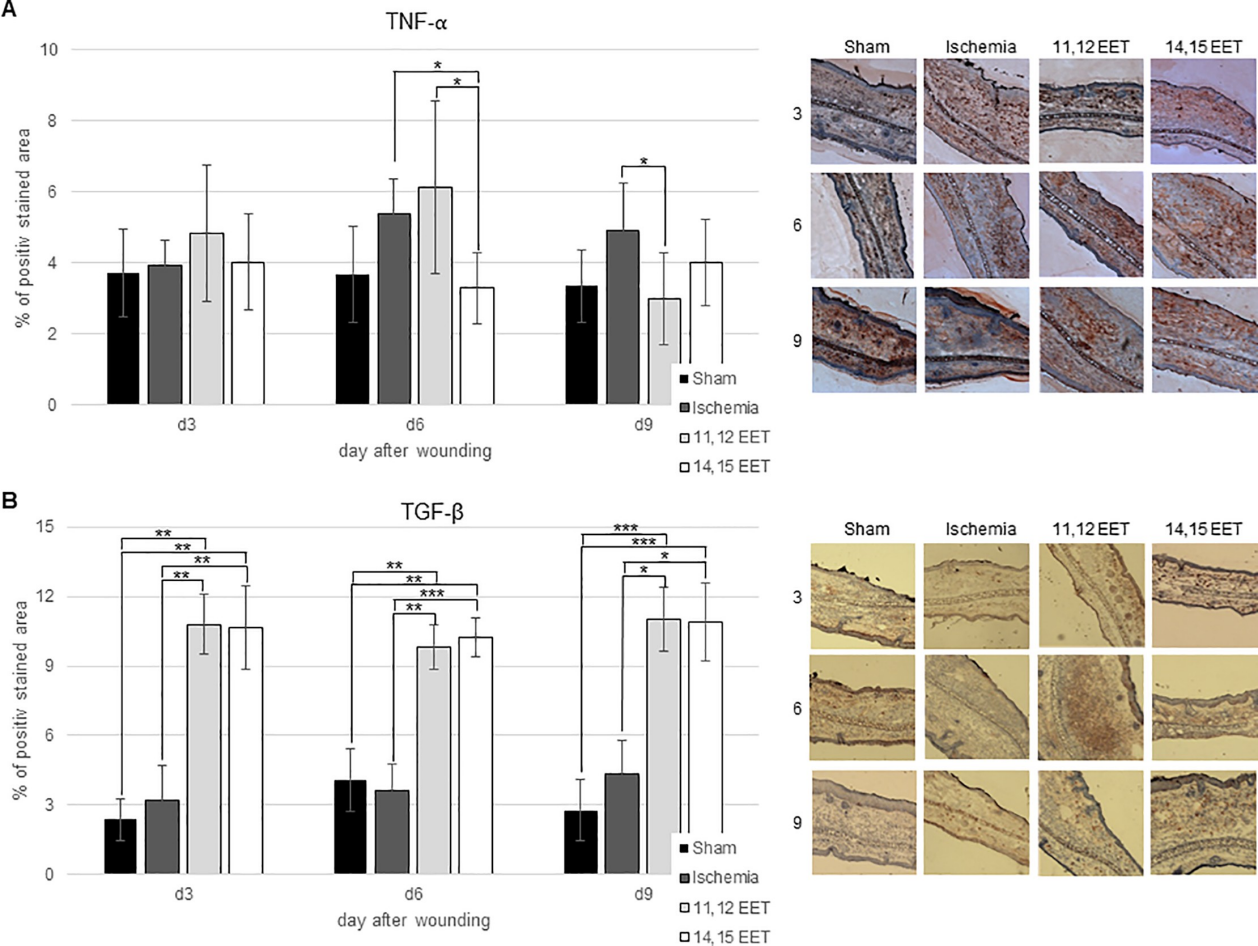
A Percentage of TNF-α positive area on day 3, 6, and 9 after wounding of control, ischemic and 11,12 as well as 14,15 EET treated ischemic wounds. On the right representative pictures of immunohistological staining. B Percentage of TGF-β positive area on day 3, 6 and 9 after wounding of control, ischemic and 11,12 as well as 14,15 EET treated ischemic wounds. On the right representative pictures of immunohistological staining (data is shown as mean ± SD; *n = 8*). *p<0.05, ***p<0.001.

Expression of TNF-α seemed to be enhanced on day 6 and day 9 in ischemic wounds compared to control though this finding was not significant (Fig 4A). This suggests a prolonged inflammatory state in ischemic wounds.

After treatment with 11,12 EET expression of TNF-α appeared to be elevated on day 3 and day 6 after wounding compared to control, ischemia and 14,15 EET treatment though this was only significant on day 6 compared to 14,15 EET. TNF-α level normalized to control group on day 9 and was significantly lower than in the ischemic group on this day (Fig 4A). So initial raised inflammatory reaction by TNF-α receded to normal in later stages of wound healing. TNF-α expression in wounds of 14,15 EET treated mice was comparable to control ears throughout the whole period of wound healing and was significantly lower on day 6 compared to ischemic wounds and 11,12 EET treatment (Fig 4A).

TGF-β expression in ischemic wounds equalled that of control group on day 3 and day 6 and appeared to be elevated on day 9 compared to control (Fig 4B). This supports the thesis of a prolonged inflammatory reaction under ischemic conditions.

Treatment with both 11,12 EET and 14,15 EET raised TGF-β expression in wounds throughout the whole period of wound healing significantly to control group and also to ischemic group (Fig 4B). Between 11,12 EET and 14,15 EET application no difference could be observed (Fig 4B). Thus indicating that especially the strongly elevated expression of TGF-β might be key to ameliorated wound healing after local application of EET.

**Fig 5:**
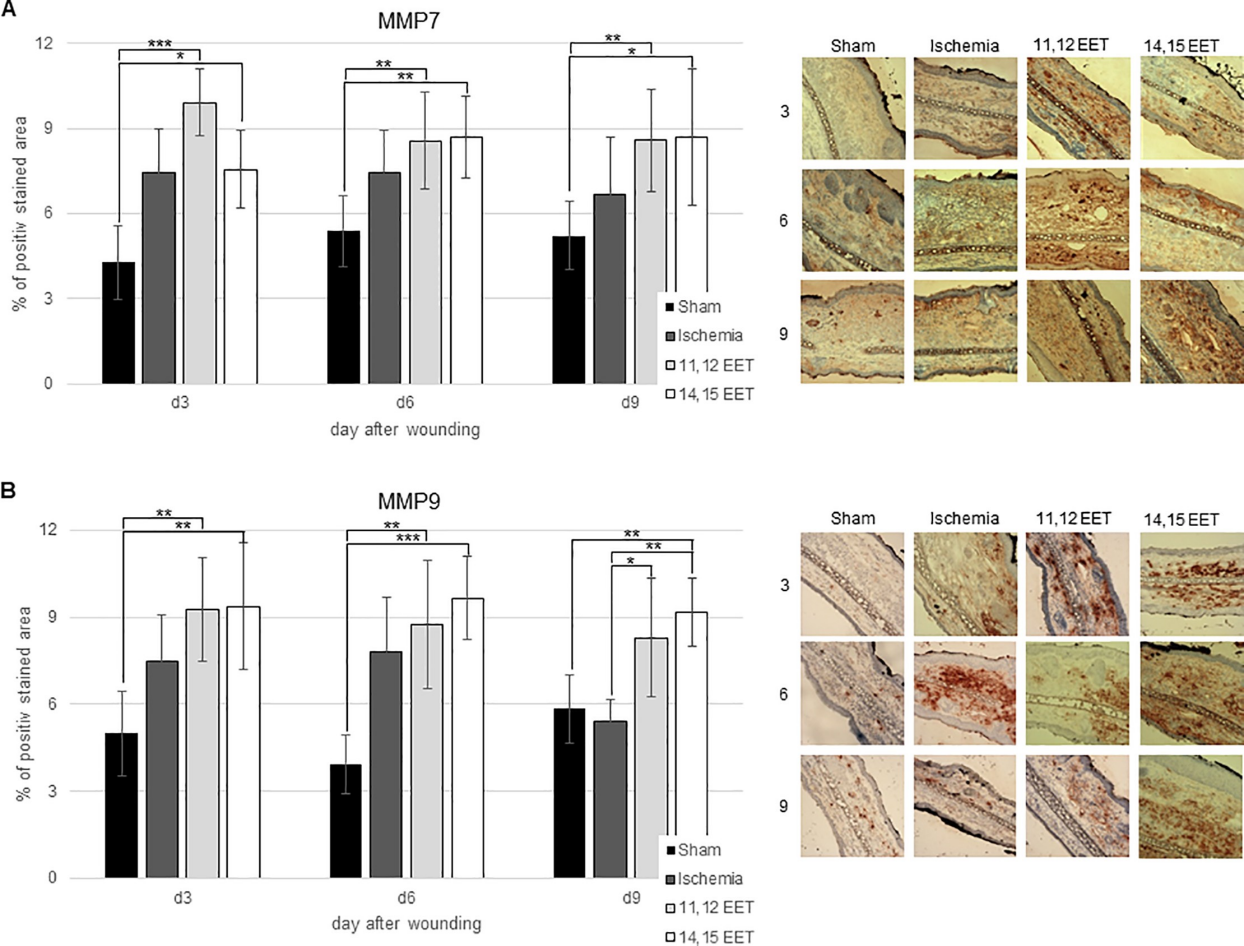
A: Percentage of MMP7 positive area on day 3, 6 and 9 after wounding of control, ischemic and 11,12 as well as 14,15 EET treated ischemic wounds. On the right representative pictures of immunohistological staining. B: Percentage of MMP9 positive area on day 3, 6 and 9 after wounding of control, ischemic and 11,12 as well as 14,15 EET treated ischemic wounds. On the right representative pictures of immunohistological staining (data is shown as mean ± SD; *n = 8*). *p<0.05, **p<0.01, ***p<0.001.

**Fig 6:**
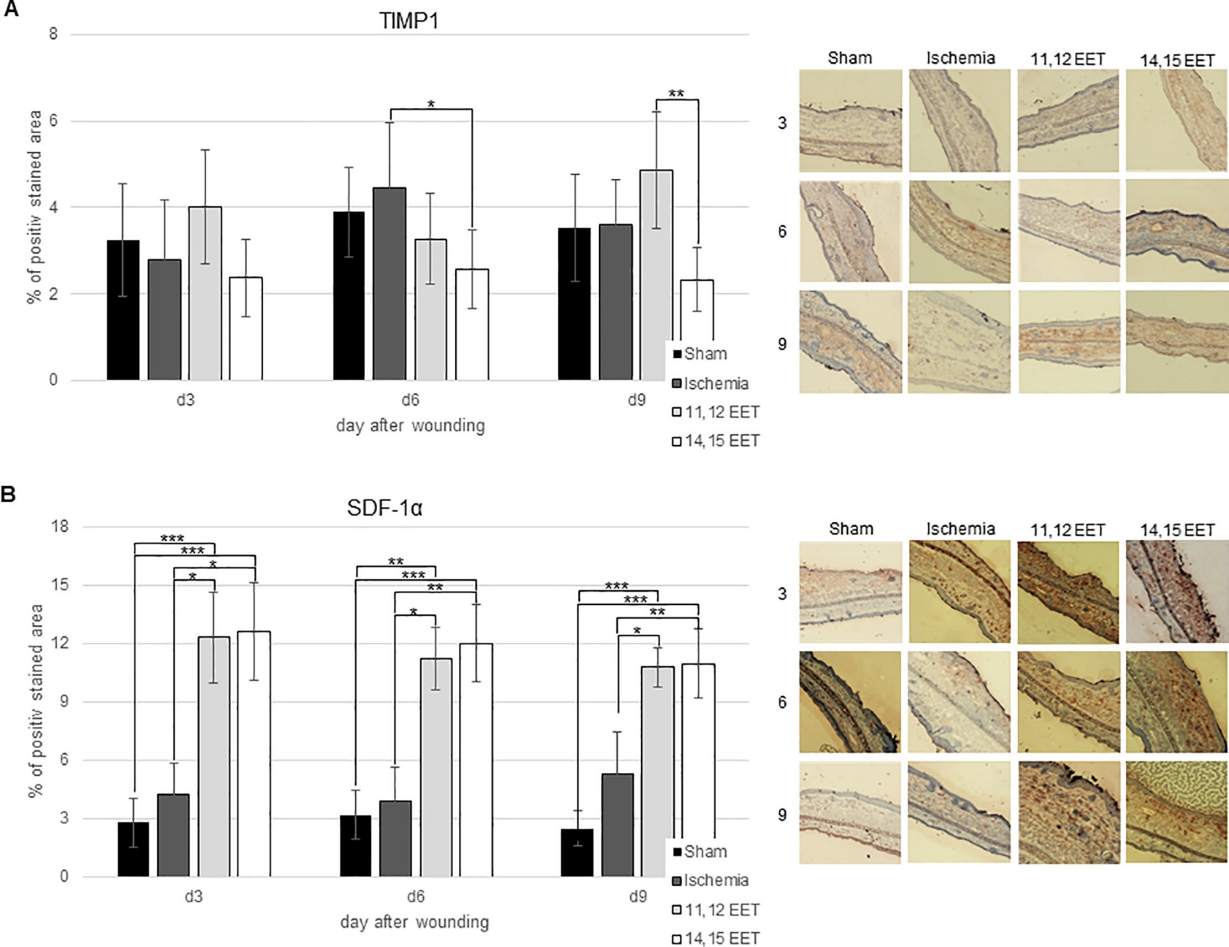
A: Percentage of TIMP1 positive area on day 3, 6 and 9 after wounding of control, ischemic and 11,12 as well as 14,15 EET treated ischemic wounds. On the right representative pictures of immunohistological staining. B: Percentage of SDF-1α positive area on day 3, 6 and 9 after wounding of control, ischemic and 11,12 as well as 14,15 EET treated ischemic wounds. On the right representative pictures of immunohistological staining (data is shown as mean ± SD; *n = 8*). *p<0.05, **p<0.01, ***p<0.001.

Local level of MMP7 in ischemic wounds seemed to be higher throughout the whole wound healing period compared to control, though this was no significant (Fig 5A). Comparably MMP9 level appeared to be raised in ischemic wounds on day 3 and 6 compared to control though also not significantly (Fig 5B). On day 9, MMP9 expression in ischemic wounds equalled the expression in control group (Fig 5B). In contrast to this, ischemia did not alter TIMP1 level during the whole period of wound repair compared to control (Fig 6A).

Local application of 11,12 EET and 14,15 EET significantly raised the level of MMP7 throughout the whole period of wound healing in comparison to control and level of MMP9 on day 3 and day 6 for 11,12 EET as well as day 3, day 6 and day 9 for 14,15 EET (Fig 5A and 5B). Compared to ischemia 11,12 EET and 14,15 EET application significantly raised the expression MMP9 on day 9 (Fig 5B).

Though 14,15 EET application showed only a little influence on MMP expression compared to ischemia, it significantly lowered TIMP1 expression on day 6 compared to ischemia and on day 9 compared to 11,12 EET (Fig 6A). In contrast to this findings, 11,12 EET application seemed to enhanced level of TIMP1 expression on day 3 and day 9 compared to control and ischemia though this was not significant (Fig 6A). Thus 11,12 EET as well as 14,15 EET change expression of MMP and their inhibitor TIMP in wound healing.

SDF-1α expression in ischemic wounds appeared to be increased only on day 9 after wounding compared to control though this was not significant (Fig 6B). During earlier stages, it almost equalled the expression in control wounds. In accordance to earlier findings, 11,12 as well as 14,15 EET application enhanced SDF-1α expression throughout the whole wound healing process significantly compared to control and non-treated ischemic wounds (Fig 6B).

### Evaluation of proliferation in wounds

As ischemic wounds after treatment with EETs exhibited a significantly faster closure, evaluation of proliferation in wounds as a means for faster wound closure was evaluated by Ki67 expression in cells.

First of all, we found that in ischemic wounds proliferation was almost equal to that of non-ischemic ears throughout the whole period of wound healing (Fig 3B). This indicates that proliferation does not seem to be responsible for delayed wound healing under ischemic conditions (Fig 3B).

In contrast to this finding proliferation after treatment with 11,12 EET and 14,15 EET in ischemic wounds was significantly elevated on all days measured after wounding (Fig 3B). This finding indicates that elevated proliferation rate after treatment of wound with EETs might be one of the major reasons for enhanced wound closure and reepithelialisation.

## Discussion

In this study, we evaluated the effect of 11,12 EET as well as 14,15 EET on deteriorated wound healing under ischemic conditions. For this purpose, we used the model of the hairless mouse, where ischemia was induced by cauterization of 2 of the 3 suppling vessels as described previously [23,24]. EETs are well known to regulate inflammation, vascular tone, and neogangiogenesis [3]. Thus, we analyzed the effect of local EET application on wound closure, neovascularization, inflammation, and proliferation in the wounds.

We could confirm that ligation diminished blood supply to the wound area impairs local wound healing as it has already been described by Kamler et al. [23]. Local application of 11,12 EET and 14,15 EET could rescue deteriorated wound healing in our model of ischemic wounds as time to wound closure was reduced to normal by 11,12 EET and was only slightly longer after application of 14,15 EET compared to non-ischemic control.

Ischemia itself did not significantly increased the expression of VEGF in wounds compared to control showing that the insufficient angiogenesis might be one of the reasons for impaired wound healing in ischemia. Local application of 11,12 EET as well as 14,15 EET raised level of VEGF significantly compared to non-ischemic control and to ischemic wounds. This augmented expression alludes to the hypothesis that enhanced wound healing under ischemic conditions with locally applied EETs might be primarily explained by an increased neovascularisation supporting an sufficient blood supply to the wound.

In our hands, ischemia appeared to lead to a prolonged augmentation of TNF-α in wounds on day 6 and day 9 after wounding though this was not significant. This suggests an extended inflammatory state in ischemic wounds and supports earlier findings where the development of chronic wounds is attributed to an unresolved inflammatory state [2]. After treatment with 11,12 EET TNF-α expression seemed to be elevated on day 3 and day 6 after wounding compared to non-ischemic control but normalized during the later stages of wound healing. In contrast to this, application of 14,15 EET did not change TNF-α level compared to non-ischemic control wound.

Thus, 11,12 EETs seem to enhance initial inflammatory reaction that resolves in later stages to normal. This change in TNF-α expression might also be a key in amelioration of healing. This early elevation of TNFα is not observed in 14,15 EET treated wounds that exhibit a poorer wound healing than those after 11,12 EET treatment. Accordingly, we recently observed reduced initial inflammatory reaction was associated with prolonged wound healing during sepsis [25]. Furthermore, local 11,12 EET treatment reduced TNF-α expression in late stages of wound healing compared to untreated ischemic wounds normalizing inflammatory reaction to non-ischemic control. These finding support the notion that prolonged inflammation in wounds promotes the formation of chronic wounds as suggested earlier [2].

No difference in TGF-β expression was observed between ischemic and non-ischemic wound throughout the whole wound healing process. But local application of 11,12 EET and 14,15 EET raised the TGF-β level on all days measured. TGF-β is one of the most important factors for successful wound healing as it is a powerful chemoattractant for monocytes and macrophages as well as fibroblasts [26]. It also induces angiogenesis and positively regulates ECM production by fibroblasts. Overexpression of TGF-β also induces a faster epithelialization of wounds though this role of the cytokine is yet to be discussed [27]. Nevertheless, elevated TGF-β expression after EET application might be another key impact positively influencing wound healing under ischemic conditions.

Function and release of cytokines as well as angiogenesis, epithelialization and scar formation are also regulated by MMPs and their inhibitors [16]. Ischemia did not significantly change MMP7 or MMP9 expression in wounds though it seemed to be elevated during the early period of wound healing compared to control. 11,12 and 14,15 EET application increased level of MMP7 and of MMP9 during the all stages of healing. MMPs are able to inflict multiple different functions in the wound. First, MMPs regulate inflammatory reaction. They can enhance inflammation by cleaving pro-inflammatory cytokines from the cell surface [13]. They can also undermine inflammation by degrading the same cytokines. Furthermore, MMPs can cleave different components of cell-cell junctions and in this way promote reepithelialization. They also take part in creating a chemotactic gradient by degradation or activation of different chemokines. For example, MMP7 is a key regulator for transepithelial neutrophil migration after lung injury [28]. Thus, elevation of MMPs might be part of enhanced neovascularization, epithelialization and change in inflammation leading to an ameliorated healing by EETs.

Furthermore, we analyzed SDF-1α expression. SDF-1α is an important chemokine for homing of stem and progenitor cells and in this way positively influences healing [20]. After induction of ischemia, no significant elevation of SDF-1α was found though it appeared to be augmented on day 9 when comparing control. This might indicate the prolonged healing process as there is a prolonged enhanced expression of SDF-1α. SDF-1α expression was highly elevated in wounds after application of 11,12 and 14,15 EET, confirming the results that were found earlier under non-ischemic conditions [4]. Thus, enhanced wound healing under ischemic conditions by EETs is at least partially mediated by increased SDF-1α. SDF-1α influences also keratinocyte migration and proliferation positively which might lead to an ameliorated epithelialization and in this way improves wound closure [20,21].

Finally, evaluation of keratinocyte proliferation showed only a small difference between ischemic and non-ischemic wounds on day 6 that was not even significant, suggesting that proliferation does not seem to be responsible for delayed wound healing under ischemic conditions. On the other hand, treatment with 11,12 and 14,15 EET elevated amount of proliferating cells significantly throughout the whole period of wound healing. This effect can be partially explained by elevated SDF-1α found in EET treated wounds and in this way confirm the in vitro results found by Florin et al. [21]. In the wound model we used wound closure is facilitated by reepithelialisation as wound contraction is inhibited by the adhesion of the epithelium to the underlying cartilage of the mouse ear. Thus, enhanced proliferation might be one of the key factors for faster wound closure after local EET application.

In summary, 11,12 and 14,15 EET improve delayed wound healing in ischemia. This effect is mediated by enhanced proliferation and neovascularization as well as an alteration of inflammation in wounds.

## Supporting information

S1 Table Raw data of wound area and day of wound closure of control, ischemic and 11,12 as well as 14,15 EET treated ischemic wounds. (XLSX)

S2 Table Raw data of percentage of VEGF positive area on day 3, 6 and 9 after wounding of control, ischemic and 11,12 as well as 14,15 EET treated ischemic wounds. (XLSX)

S3 Table Raw data of percentage of Ki67 positive area on day 3, 6 and 9 after wounding of control, ischemic and 11,12 as well as 14,15 EET treated ischemic wounds. (XLSX)

S4 Table Raw data of percentage of TNF-α positive area on day 3, 6 and 9 after wounding of control, ischemic and 11,12 as well as 14,15 EET treated ischemic wounds. (XLSX)

S5 Table Raw data of percentage of TGF-β positive area on day 3, 6 and 9 after wounding of control, ischemic and 11,12 as well as 14,15 EET treated ischemic wounds. (XLSX)

S6 Table Raw data of percentage of MMP7 positive area on day 3, 6 and 9 after wounding of control, ischemic and 11,12 as well as 14,15 EET treated ischemic wounds. (XLSX)

S7 Table Raw data of percentage of MMP9 positive area on day 3, 6 and 9 after wounding of control, ischemic and 11,12 as well as 14,15 EET treated ischemic wounds. (XLSX)

S8 Table Raw data of percentage of TIMP1 positive area on day 3, 6 and 9 after wounding of control, ischemic and 11,12 as well as 14,15 EET treated ischemic wounds. (XLSX)

S9 Table Raw data of percentage of SDF-1α positive area on day 3, 6 and 9 after wounding of control, ischemic and 11,12 as well as 14,15 EET treated ischemic wounds. (XLSX)

## Abbreviations

EET: epoxyeicosatrienoic acid
MMP7: matrix metalloproteinase 7
MMP9: matrix metalloproteinase 9
TIMP1: tissue inhibitor of matrix metalloproteinase 1
SD: standard deviation
TNF-α: tumor necrosis factor alpha
TGF-β: tumor growth factor-beta
VEGF: vascular endothelial growth factor
SDF-1α: stromal cell-derived factor 1α
